# A comparative study of the physiological and psychological effects of forest bathing (Shinrin-yoku) on working age people with and without depressive tendencies

**DOI:** 10.1186/s12199-019-0800-1

**Published:** 2019-06-22

**Authors:** Akemi Furuyashiki, Keiji Tabuchi, Kensuke Norikoshi, Toshio Kobayashi, Sanae Oriyama

**Affiliations:** 1 Institute of Biomedical and Health Sciences, Hiroshima University, 1-2-3, Kasumi, Minami-ku, Hiroshima, 734-8551 Japan; 2 Research and Education Faculty, Medical Sciences Cluster, Nursing Science Unit, Kochi University, Kohasu Okocyo, Nankoku, Kochi 783-8505 Japan; 3 Faculty of Nursing, Hiroshima International University, 5-1-1, Hiro koshingai, Kure, Hiroshima 737-0112 Japan; 4 Department of General Internal Medicine, Ishii Memorial Hospital, 3-102-1, Tada, Iwakuni, Yamaguchi 741-8585 Japan

**Keywords:** Forest bathing, Depressive tendencies, Working age, Physiological effects, Psychological effects

## Abstract

**Background:**

In recent years, many of Japanese workers have complained of fatigue and stress, considering them as risk factors for depression. Studies have found that “forest bathing” (Shinrin-yoku) has positive physiological effects, such as blood pressure reduction, improvement of autonomic and immune functions, as well as psychological effects of alleviating depression and improving mental health. In this study, we investigate the physiological and psychological effects of “forest bathing” on people of a working age with and without depressive tendencies.

**Methods:**

We conducted physiological measurements and psychological surveys before and after forest bathing with subjects who participated in day-long sessions of forest bathing, at a forest therapy base located in Hiroshima Prefecture. After excluding severely depressed individuals, the participants were classified into two groups: those with depressive tendencies (5 ≤ K6 ≤ 12) and those without depressive tendencies (K6 < 5) for comparative study. The evaluation indices measured were systolic blood pressure (SBP), diastolic blood pressure (DBP), pulse rate (PR), autonomic functions, and profile of mood states (POMS).

**Results:**

Of the 155 participants, 37% had depressive tendencies, without any differences observed between males and females. All participants showed significant decrease in SBP, DBP, and in negative POMS items after a forest bathing session. Before the session, those with depressive tendencies scored significantly higher on the POMS negative items than those without depressive tendencies. After forest bathing, those with depressive tendencies demonstrated significantly greater improvement in many of POMS items than those without depressive tendencies, and many of them no longer differed between those with and without depressive tendencies.

**Conclusions:**

Examining the physiological and psychological effects of a day-long session of forest bathing on a working age group demonstrated significant positive effects on mental health, especially in those with depressive tendencies.

Not applicable; this is not a report of intervention trial.

## Introduction

In Japan, approximately 60% of workers complain about strong feelings of anxiety, worry, and stress related to work and occupation [1], so fatigue and stress are regarded as strong risk factors for depression [2, 3]. According to the WHO’s World Mental Health Survey, about 10% of people with mild depressive symptoms develop clinical depression. The initial response to this has been identified as being important because there is a danger of more severe symptoms developing, as well as an increasing risk of suicide when symptoms are neglected [4, 5]. Many of the increased instances of depression are reported as mild depression, with sub-threshold depressive symptoms and mood disorders that do not meet the diagnostic criteria for depression, as well as non-clinical depression [6–8]. Approaches other than drug-based therapy for depression, including cognitive behavioral therapy (CBT), psychotherapy, and other interpersonal therapies, are recommended for mild depression [6–8]. The UK’s depression treatment guidelines identify that exposure to the outdoors and a forest environment promotes resilience, and aerobic exercise due to physical activities such as walking alleviates depressed states, and improves mental health for those with sub-threshold depressive symptoms [7, 9, 10]. Moreover, it has been reported that CBT in a forest environment demonstrates lower rates of recurrence of depressive symptoms and social maladjustment, and higher rates of remission than treatment in hospital [11].

The effects of exposure to a forest environment include recovery from stress [12], and alleviation of the effects of reduced attention resulting from fatigue [13]. In Japan, “forest bathing” (Shinrin-yoku) was first advocated by the Forestry Agency in 1982, identified as a form of recreation involving walking and inhaling the fragrant substances released by trees. The act of “forest bathing” has been regarded as a natural remedy that brings about improvements in terms of human physical and mental health [14]. Studies have reported relaxation and the effects on organisms arising from terpene components such as phytoncide, which are emitted from trees [15, 16]. Miyazaki [17] conducted a physiological and psychological investigation on young males in various locations across Japan, comparing the short-term effects of forest bathing, with the same in suburban areas. The study reported greater physiological effects from forests than urban areas, such as a decrease in blood pressure, the activation of parasympathetic nervous activity, and the suppression of sympathetic nervous activity, as well as biochemical effects such as decreased salivary amylase and blood cortisol concentrations, and increased immune function [18, 19]. Moreover, improvement in psychological functioning, such alleviating negative emotions and increasing positive emotions were identified [20]. Forest bathing has been demonstrated to improve negative mood using the profile of mood states (POMS) mood scale [20, 21], as well as improvements in depressive symptoms [22].

While systematic review of forest therapy pointed that forest therapy may play an important role in health promotion and disease prevention, the lack of high-quality studies limits the strength of results, rendering the evidence insufficient to establish clinical practice guidelines for its use [23]. Further, there has been insufficient consideration of the physiological and psychological effects of forest bathing on workers with high stress and depressive tendencies. In particular, there have been a few studies that have used depression scales to examine changes in depressive tendencies [24–26]. Since depressive tendencies that do not meet the criteria for depression indicate a higher risk of becoming depression [5, 8], it is important to consider the effect of forest bathing on Japanese workers with high stress and depressive tendencies to prevent the deteriorating mental health of working age people.

In this study, a group of working age people who participated in forest bathing were classified into two groups based on the presence or absence of depressive tendencies, to conduct an investigation into the physiological and psychological effects of forest bathing, and compare and examine any changes observed.

## Methods

### Participants

In the day-long sessions of forest bathing, which were held a total of 16 times during the 3-year period from October 2012 to November 2014 at the Akiota town Forest Therapy Base in Hiroshima Prefecture, the information on forest bathing was distributed to working age people, living in the Hiroshima city areas, who mainly worked in companies. Then, 219 people applied and their written consent was acquired for this study.

Inclusion criteria for the participants were working people aged between 18 and 60. Exclusion criteria were pregnant women, suspicion of severe depression (13 ≤ K6), and history of depressive and/or cardiovascular diseases. Out of the total participants, 165 participants (19–59 years old) consented to participate in this study. Ten participants who had a history of cardiovascular disease or mental illness, or who were undergoing medical treatment at the time were excluded. The rest of the 155 participants (94%) were included in the analysis. Eighty-seven of these also participated in the evaluation of autonomic nervous activity, who were assessed before and after forest therapy.

### Experimental design

The forest therapy base used for our field research is located within a national park in a valley in the region around the source of the Ota River, in the northwestern part of Hiroshima Prefecture with mountains in the 1000 m range. The vegetation mainly comprises mixed natural forests, with a temperate climate typical of the Setouchi region. The Ryuzukyo Forest Therapy Road used in this study is 3.5 km in length and 335–490 m in altitude (altitude difference of 155 m).

**Fig. 1:**
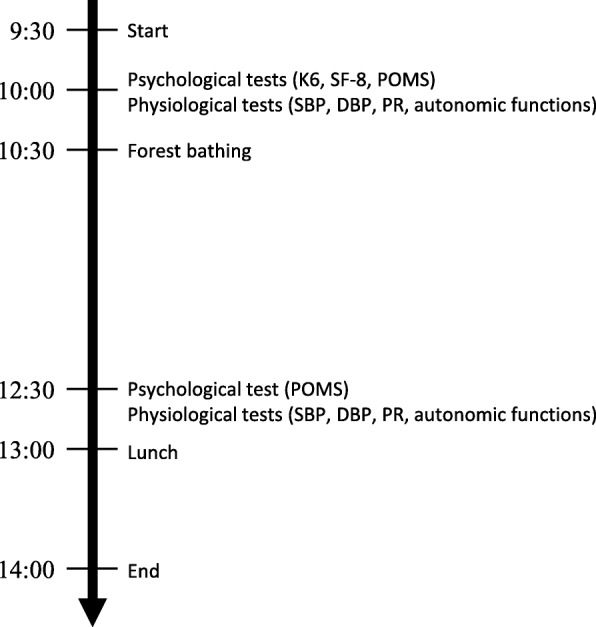
The protocol for forest bathing

Figure 1 shows the protocol for forest bathing as follows: participants were gathered at 9:00 am, the nature of the research was explained, and psychological surveys and physiological measurements were conducted after gaining consent from the participants. Participants were placed into groups of either four or five people for forest bathing, which involved slowly walking around the forest for about 2 h, initially with one or two guides. In addition to explaining the natural environment of the forest, the guides demonstrated breathing methods, yoga, hammock experiences, etc. at each point, encouraging communication among the participants throughout the forest bathing. After forest bathing, the same surveys and measurements were conducted again. All the participants carried out forest bathing in the autumn (86.5%) or spring (13.5%) season. The weather was generally sunny or cloudy, with a temperature of 12–25 °C and humidity of 40–80% without any environmental noise. The participants walked 5710 ± 620 steps on average during 2-h forest bathing, consuming 337–445 kcal.

This research was registered by the Ethics Committee of Hiroshima University (H25–29 30/04/2014).

### Evaluation scales and measurement methods

#### Psychological measures

Psychological distress was measured using the Kessler Psychological Distress Scale K6 [4, 5, 27] which evaluates six items on a scale ranging from (0) “not at all” to (4) “always,” with a maximum total score of 24 points. The evaluation of depressive tendencies by K6 score is set in relation to certain cutoff points; a K6 score of 13 or more indicates that mental health treatment is required, while the cutoff point for mental distress is a K6 score of 5 or more [25, 28]. In this study, those whose K6 score was 13 or more were excluded, and participants were classified into two groups based on depressive tendencies: those with depressive tendencies (a K6 score of 5 to 12 inclusive) and those without depressive tendencies (a K6 score of 4 or less).

Health-related quality of life was measured with SF-8 [29], which comprises eight items as follows: general health, physical functioning, physical roles, bodily pain, vitality, social functioning, mental health, and emotional roles. It also evaluates a physical component summary (PCS) and mental component summary (MCS). These are standardized as Japanese national standard values of 50 points and their standard deviations are 10 points. A higher score indicates better health [29].

Emotional profiles were measured with the Japanese edition of POMS [21]. This scale comprises 30 items in six subscales as follows: Tension-Anxiety (T-A), Depression-Dejection (D-D), Anger-Hostility (A-H), Fatigue (F), Confusion (C), and Vigor (V). T-scores standardized by age and sex were calculated. Additionally, the total mood disturbance (TMD) score was calculated [TMD = (T-A) + (D-D) + (A-H) + F + (C-V)].

#### Physiological measures

The circulatory functions of systolic blood pressure (SBP), diastolic blood pressure (DBP), and pulse rate (PR) were measured using an oscillometric monitor (Omron HEM-1011, Omron Healthcare, Tokyo, Japan). Autonomic nervous activities were measured using a finger-tip volumetric pulse wave meter (Pulse Analyzer Plus View TAS-9, YKC, Tokyo, Japan) [30]. Using the pulse meter waveform synchronized with the heartbeat as an index, a fast Fourier transform and spectrum analysis were performed to measure the power of low frequency (LF: 0.04–0.15 Hz) components and high frequency (HF: 0.15–0.4 Hz) components. HF was used as an indicator of parasympathetic activity, and LF/HF was used to assess the balance between sympathetic and parasympathetic nervous activity [31].

Circulatory function (SBP, DBP, PR), autonomic nerve function, and POMS were examined and compared before and after the forest bathing session. Additionally, the participants were classified into two groups according to the presence or absence of depressive tendencies, and a comparison was made between the two groups before and after the forest bathing session.

This study was conducted with the approval of the Department of Nursing Development Science Ethics Review Committee of the Integrated Health Sciences, Institute of Biomedical and Health Sciences at Hiroshima University (approval numbers: H 24–27 and H 25–29). We explained that cooperation with the study was based on participants’ free will and their cooperation could be withdrawn even after consent to participate in the study had been provided. Additionally, since forest bathing is conducted in a natural environment, medical staff also participated in forest bathing session to attend to any emergency.

### Statistical analysis

The 155 participants’ circulatory function and POMS were compared and analyzed before and after a forest bathing session. Changes in autonomic functions were also compared in the 87 subjects who were measured correctly. Participants were also classified into two groups according to the presence or absence of depressive tendencies, and a comparison of the two groups was conducted. During the analysis, the correlation between SF-8 and K6, and the normality of data were confirmed using a Shapiro–Wilk test. Then, *t* tests, a Wilcoxon signed rank test, a Chi-square test, a Mann–Whitney *U* test, simple regression analysis, and Spearman’s rank correlation coefficient were used (SPSS ver. 24). The value for significance was set at *p* < 0.05.

## Results

### Summary of participants

As Table 1 demonstrates, the 155 participants encompassed an age range of 19–59 and their mean age was 44.0 ± 3.2. The participants were classified into four groups: those under 30, those in their 30s, those in their 40s, and those in their 50s. Further, 55.5% of the subjects were female (Table 1).

**Table 1:** Characteristics of the subjects

	All subjects (*n* = 155)		Non-depressive tendency^a^ (*n* = 97)	Depressive tendency^b^ (*n* = 58)	*P* value^c^
	Mean ± SD	*n* (%)	*n* (%)	*n* (%)	
Age	44.0 ± 9.6	155 (100)	45.0 ± 9.7	42.3 ± 9.3	0.470
Age groups					
≤ 29	5.33 ± 3.60 (K6 score)	12 (7.7)	5 (41.7)	7 (58.3)	0.068
30–39	3.40 ± 3.27 (K6 score)	40 (25.8)	26 (65.0)	14 (35.0)	
40–49	4.35 ± 3.28 (K6 score)	52 (33.5)	28 (53.8)	24 (46.2)	
50–59	2.76 ± 2.83 (K6 score)	51 (32.9)	38 (74.5)	13 (25.5)	
Sex					
Male	3.68 ± 2.86 (K6 score)	69 (44.5)	41 (59.4)	28 (40.6)	0.507
Female	3.64 ± 3.52 (K6 score)	86 (55.5)	56 (65.1)	30 (34.9)	
Body mass index	22.3 ± 3.2	101 (100)	22.5 ± 2.9	22.0 ± 3.4	0.816
Medication					
no	3.45 ± 3.23 (K6 score)	127 (81.9)	84 (66.1)	43 (33.9)	0.056
yes^d^	4.51 ± 3.17 (K6 score)	28 (18.1)	13 (46.4)	15 (53.6)	
Health-related QOL					
PCS	51.2 ± 4.5	155 (100)	51.3 ± 3.8	51.1 ± 5.5	0.916
MCS	47.9 ± 6.0	155 (100)	50.6 ± 4.7	43.2 ± 5.0	< 0.001
K6 score	3.66 ± 3.24	155 (100)	1.56 ± 1.50	7.17 ± 2.10	< 0.001

*QOL* quality of life, *PCS* physical component summary, *MCS* mental component summary

^a^Non-depressive tendency: K6 ≤ 4

^b^Depressive tendency: 5 ≤ K6 ≤ 12

^c^Chi-square test or Mann–Whitney *U* test (non-depressive tendency vs. depressive tendency)

^d^Medication: hypertension (*n* = 6), dyslipidemia (*n* = 5), diabetes mellitus (*n* = 3), others (*n* = 14)

The mean K6 score was 3.66 ± 3.24. Fifty-eight participants (37.4%) evinced depressive tendencies (K6 of 5–12) and 97 participants (62.6%) did not demonstrate depressive tendencies (K6 of 4 or less). The mean values of K6 score for those with depressive tendencies by age were also shown in Table 1. Those under 30 years old and those in their 40s exhibited higher depressive tendencies. However, there was no significant difference between age groups. The proportions and mean values for those with depressive tendencies by sex were 40.6% of males, with an average of 3.68 ± 2.86 points, and 34.9% of females, with an average of 3.64 ± 3.52, which was no significant difference in depressive tendencies in terms of gender.

The health-related quality of life (QOL) mean SF-8 scores for all 155 participants revealed that the PCS score of 51.2 ± 4.5 was slightly higher than the average compared to the Japanese standard value of 50. On the other hand, the MCS was 47.9 ± 6.0, slightly lower than the average (Table 1). Additionally, while there was no significant difference between those with depressive tendencies and those without depressive tendencies in terms of PCS, the MCS scores were significantly lower (*p* < 0.001) for the depressive group. There was also no significant difference between the PCS and MCS scores in the group without depressive tendencies, but the MCS score was significantly lower (*p* < 0.001) than the PCS score in the depressive tendencies group. The values obtained from the depressive tendencies group were significantly lower than those obtained from the group without depressive tendencies (*p* < 0.05 to *p* < 0.001) for all measures of the QOL subscales, apart from physical functioning (data was not shown).

### Changes in physiological indicators based on depressive indicators for all participants and for the two groups

Changes in circulatory function before and after forest bathing were assessed by measuring the blood pressure of all subjects, revealing that SBP was 129.8 ± 20.4 mmHg before forest bathing and 121.5 ± 19.3 mmHg after the activity was undertaken (Table 2). DBP was 79.0 ± 15.0 mmHg before forest bathing and 74.6 ± 13.8 mmHg after the activity. Hence, all participants recorded a significant decrease in both SBP and DBP (*p* < 0.001) after the forest bathing. Measurements of autonomic nervous function revealed that neither parasympathetic nerve activity (ln HF) nor sympathetic nerve activity (ln LF/HF) registered any significant change.

**Table 2:**
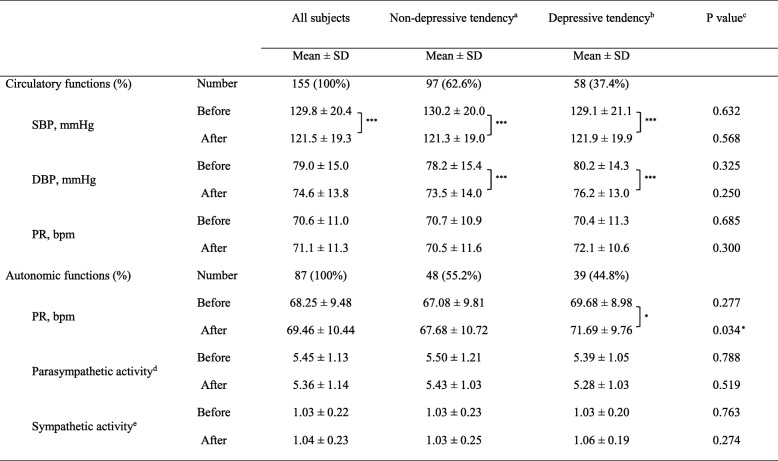
Changes of circulatory functions and autonomic functions by Forest bathing in the subjects with and without depressive tendencies

*SBP* systolic blood pressure, *DBP* diastolic blood pressure, *PR* pulse rate

*p < 0.05, ***p < 0.001, paired *t* test (before vs. after)

^a^Non-depressive tendency: K6 ≤ 4

^b^Depressive tendency: 5 ≤ K6 ≤ 12

^c^Wilcoxon signed–rank test (non-depressive tendency vs. depressive tendency)

^d^Logarithmically transformed value of HF

^e^Logarithmically transformed value of LF/HF

A comparison of the data for the depressive tendencies group and without depressive tendencies group before and after the forest bathing yielded no difference in blood pressure, in either SBP or DBP levels. Both groups registered a significant decrease in blood pressure, both SBP and DBP after forest bathing (*p* < 0.001). However, the depressive tendencies group exhibited a significant rise (*p* < 0.05) in the PR after forest bathing, and this increase was notably higher than the escalation found in the without depressive tendencies group (*p* < 0.05). Additionally, no significant differences were found between the two groups for ln HF and ln LF/HF before and after forest bathing (Table 2).

### Changes in psychological indicators for all participants and for the two groups based on the presence or absence of depressive tendencies

After forest bathing, the POMS scores for all participants in the five negative subscales decreased significantly: Tension-Anxiety (T-A); Depression-Dejection (D-D); Anger-Hostility (A-H); Fatigue (F); and Confusion (C) (*p* = 0.001 to *p* < 0.001) (Table 3). Additionally, the TMD recorded a negative mood state at 5.8 ± 12.7 before forest bathing, and changed to a positive mood state at − 4.6 ± 6.3 after forest bathing (*p* < 0.001).

**Table 3:**
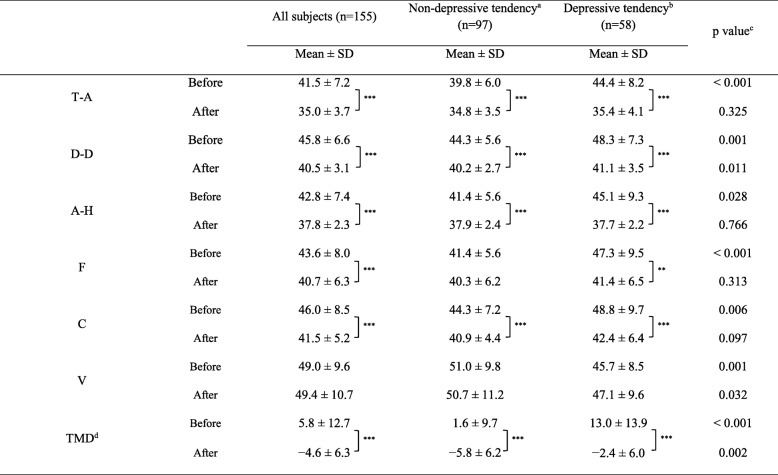
Changes in POMS and TMD scores before and after Forest bathing in the subjects with and without depressive tendencies

*T*-*A* tension-anxiety, *D*-*D* depression-dejection, *A*-*H* anger-hostility, *F* fatigue, *C* confusion, *V* vigor, *TMD* total mood disturbance

**p < 0.01, ***p < 0.001, paired *t* test (before vs. after)

^a^Non-depressive tendency: K6 ≤ 4

^b^Depressive tendency: 5 ≤ K6 ≤ 12

^c^Wilcoxon signed–rank test (non-depressive tendency vs. depressive tendency)

^d^TMD Score = (T-A) + (D-D) + (A-H) + F + C – V

**Fig. 2:**
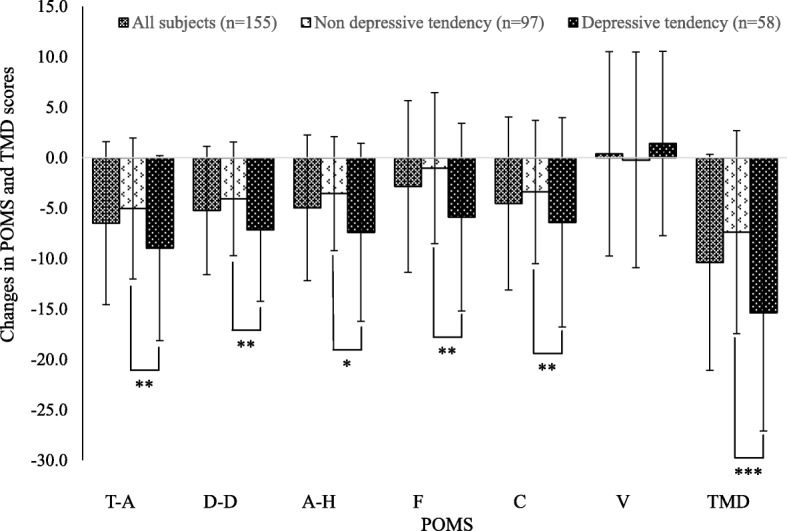
Changes in POMS and TMD scores by forest bathing (after-before value). *T-A* Tension-Anxiety, *D-D* Depression-Dejection, *A-H* Anger-Hostility, *F* Fatigue, *C* Confusion, *V* Vigor, *TMD* Total Mood Disturbance. *p < 0.05, **p < 0.01, ***p < 0.001, paired t-test

When comparing the depressive tendencies group and the without depressive tendencies group, the former evinced significantly higher negative mood subscales (T-A, D-D, A-H, F, and C) before forest bathing than the latter (*p* < 0.05 to *p* < 0.001). The positive mood subscale (V) was also significantly lower in the depressive tendencies group (*p* = 0.001). After forest bathing, both the depressive tendencies group and the without depressive tendencies groups demonstrated a significant decrease in their scores on the negative subscales, but the degree of improvement was largest for those with depressive tendencies. In fact, after forest bathing, the multiple negative scales (T-A, A-H, F, and C) for the depressive tendencies group decreased to levels equal to those recorded by the without depressive tendencies group, to the extent that any significant difference between the groups disappeared. Meanwhile, TMD measurements revealed that negative mood states were significantly greater (*p* < 0.001) before forest bathing in the depressive tendencies group than in the without depressive tendencies group, and this score decreased significantly after forest bathing. Additionally, the degree of modification in the POMS scales before and after forest bathing was computed as T-A (− 8.97), D-D (− 7.16), A-H (− 7.40), F (− 5.88), and C (− 6.41) for the depressive tendencies group; and T-A (− 5.03), D-D (− 4.09), A-H (− 3.56), F (− 1.03), and C (− 3.40) for the without depressive tendencies group. Thus, the changes observed in the depressive tendencies group were significantly larger than those witnessed in the without depressive tendencies group (*p* < 0.05 to *p* < 0.001) (Fig. 2). The TMD modification was also significantly greater in the depressive tendencies group (*p* < 0.001).

## Discussion

### Depressive tendencies of participants and health-related QOL

Of the 155 participants in this study, 37% presented with depressive tendencies (K6 of 5–12 inclusive), with the greatest number among younger and middle-aged people. For health-related QOL (SF-8), the PCS was close to the average (50 points), but the MCS was below average. According to the results of the WHO’s World Mental Health survey, and the World Mental Health Japan Survey, one in four people reported that they had experienced some kind of mental health problem in their lifetime [5]. Depressed individuals requiring treatment (K6 of 13 or more) comprised 2.7% of the sample, while 31.3% evinced depressive tendencies (K6 of 5–12) [4, 28]. The proportion of depressed individuals according to the CES-D (a score of 16 or more) was 30.6%, indicating that the results of the K6 are equivalent to those of the CES-D [25, 32]. In this study, the PCS before forest bathing for those with depressive tendencies was 51.1 and the MCS was 43.2; therefore, it appears that the level of physical health-related QOL in this group was about average, while psychological health-related QOL was lower than average.

### Changes to physiological indicators as a result of forest bathing

SBP and DBP of the participants showed a significant decrease as a result of the forest bathing for about 2 h. However, no change was observed in PR, HF, and LF/HF, which are indicators of autonomic nervous activities. This contrasts with an observational study that was conducted over 2 days in several forest regions throughout Japan [18, 19]. This study of young males walking and sitting for short periods reported the physiologically relaxing effects of walking in forested areas compared to urban cities. These results were associated with reduced SBP and DBP, as well as decreased HR and LF/HF, and an increase in HF [17, 19, 22]. However, research into the effects of multiple strolls before and after walking for 2 to 3 h in a forest environment over the course of a day revealed that although SBP decreased, DBP and PR did not change, and autonomic nervous activity was not measured [33]. The drop in blood pressure reported in the present study was the same as in previous studies, and this outcome may result from the physiologically relaxing effect derived from exposure to fragrance from the trees, and the bodily sensations of being in the forest [15–17]. It is also possible that changes in PR and autonomic nervous activities were not observed in the present study due to the difference in the age of participants, and the time they spent sitting [14, 34]. Additionally, this study observed single sessions of forest bathing of working age people, which lasted about 2 h. It is possible that participants did not reach a satisfactory state of relaxation due to the effects of physical activity during forest bathing [35].

Furthermore, no significant difference in circulatory function and autonomic nervous function was observed before forest bathing between participants with and without depressive tendencies. Both SBP and DBP showed a significant decrease in both groups after forest bathing, and only those with depressive tendencies showed a significant rise in PR. Other studies have found that those with depressive tendencies are less active in their daily lives and that they engage in fewer leisure activities [36]. This lack of activity lowers their physical fitness [37], and fatigue due to walking may reduce parasympathetic nervous activity [38]. In the present study, it is possible that the physical load and feelings of fatigue were greater for those with depressive tendencies, even only slow walking over about 2 h.

### Changes to psychological indicators as a result of forest bathing

Changes in psychological indicators (POMS), including the negative mood states (T-A, D-D, A-H, F, and C) and TMD scores were significantly improved for all participants following forest bathing. However, no alteration to the positive mood state (V) was observed. On this issue, studies of short-term walking over a period of 2 days and one night conducted in multiple areas took measurements when participants were walking or engaging in seated viewing [14, 21]. These investigations reported a significant decrease in T-A, D-D, AH, F, and C, and a noteworthy increase in V in forested areas compared to urban locations [17, 20, 22]. Additionally, improvements to negative mood due to reduced negative scales and TMD in POMS were observed, and there was only a slight increase in positive mood (V) after multiple forest bathing activities were undertaken, such as walking for 1–2 h in forested areas [33, 34]. In contrast to this, Ulrich found that emotions such as fear, anger, and disgust initially appeared in the bodies and expressions of humans upon contact with nature, followed by a prompt reaction of recovery from these negative emotions. After the negative emotions dissipated, positive emotions such as joy were subsequently found to gradually increase [12]. The present study’s findings are similar to those mentioned above: there were improvements in the negative mood scales but not in the positive scales after forest bathing. This outcome suggests that similar changes to POMS due to forest bathing can be obtained even if the ages and walking times of participants differ across studies [14, 20]. However, it has been suggested that the expression of a positive mood after forest bathing may be affected by the waking time and background of participants.

In this study, significant changes in psychological indicators were greater in those with depressed tendencies than in those without depressive tendencies. Correspondingly, the improvement recorded after forest bathing was also greater for the depressive tendencies group, to the extent that after forest bathing, there was no significant difference in negative mood state values recorded for the two groups.

**Fig. 3:**
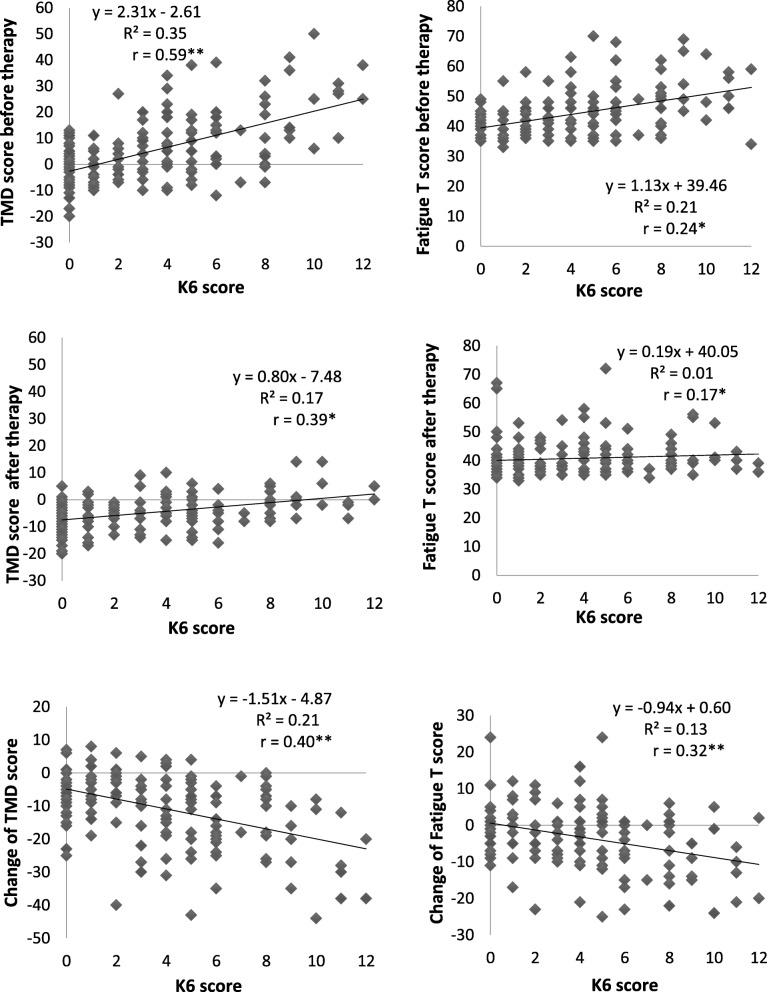
Correlation coefficient between K6 and TMD score, fatigue T score (POMS subscale). *p < 0.05, **p < 0.01, Spearman’s rank correlation co-efficient, and their changes before and after forest therapy

The POMS values before and after forest bathing were individually examined, as were the K6 scores, and the relationship between changes recorded before and after forest bathing. Figure 3 illustrates the relationship between the TMD scores, the fatigue (F) score of the subscale, and the K6 score. Before forest bathing, both the TMD and F scores showed an upward trend as K6 scores increased. After forest bathing, both TMD and F scores converged to a nearly constant range regardless of K6 scores, such that higher K6 scores were associated with a greater decrease in TMD and F scores as a result of forest bathing. Thus, a negative correlation was found between the change in TMD and F scores before and after forest bathing, and K6 scores. In this study, higher K6 values were associated with greater improvements to the TMD scores of the negative POMS mood states after forest bathing, and also linked to an improvement (decrease) of the *t* values of the negative scales. Studies have found that individuals with high blood pressure or high stress levels are more likely to exhibit improvement after forest bathing than healthy individuals [24, 34]. In this context, Damasio defines homeostasis as a state that is maintained within a preset range [39] and posits that homeostasis has two aspects: physiological and psychological. Thus, improvements in POMS negative emotions in those with depressive tendencies may have occurred due the action of psychological homeostasis. Meanwhile, Kaplan’s [13] study of the improvement of depressive tendencies through CBT incorporated the practice of mindfulness in a forest environment. This study revealed that the act of focusing one’s attention on the forest through meditation and breathing facilitated an attention recovery process, where feeling contented in one’s body and becoming aware of one’s inner self led to the acquisition of the ability to self-heal. Okamoto [2] and Yoshimura [40] reported the alleviation of depressive symptoms and psychosocial dysfunctions through a CBT intervention, as well changes to brain regions observed using functional image analysis methods. It is possible that CBT may effect changes in both the neural basis of negative cognitive-emotional interactions and in the higher order mental functions of cognition and behavior [2]. In the current study, participants mentioned positive experiences with guides and colleagues following forest bathing. Improvements observed in depressive symptoms and psychosocial functions may be linked to the possible activation of neural networks through positive emotional reflection [32], and improved social cognitive functions through the stimulation of mirror neurons, leading to a better understanding of the intended actions and emotions of others [41].

Previous study also reported that psychological and social stimulation occurs through interaction with the environment [23, 42]. Participants with depressive tendencies in this study became relaxed, and negative emotions that were strong before forest bathing were greatly reduced after forest bathing activity. This outcome confirms the physiological and psychological health benefits of forest bathing and implies potential stress reduction and recovery from fatigue for those of working age with depressive tendencies. Additionally, this study demonstrates that the use of a forest environment can enhance recovery from stress and fatigue in all participants, regardless of whether they evince depressive tendencies. Hence, forest bathing is an activity that can assist in the prevention of depression and stress-related health problems, leading to improvement in the mental health of working individuals.

However, it is possible that “regression to the mean” have influenced the results of this study demonstrating significantly greater improvement in many of POMS score for those with depressive tendencies. It is important that the improvement in POMS score for those with depressive tendencies was much greater than for the non-depressive tendencies after forest bathing, and that no more significant differences were identified in many of POMS scores between the two groups. Hence, we think that it is difficult to explain the greater improvement of POMS score in depressive tendencies simply by “regression to the mean.”

Moreover, as we did not measure sex hormones in this study, we cannot discuss the influence of the menstrual cycle of the female subjects. However, there is no difference in the ratio of males and females among the two groups. Additionally, the menstrual cycle is randomly assigned to the two groups, and no intentional bias is recognized. As this study focused on short-term changes such as examining physiological and psychological changes before and after forest bathing for individuals, it is considered that sex hormones may have little influence on the results of this study.

In this study, the improvement of mental health was statistically significant, especially in the participants with depressive tendencies. Changes were sufficiently large to be clinically meaningful. However, it will be necessary to conduct further studies to validate the efficacy of forest bathing for working age people with depressive tendencies, since other outcomes, such as autonomic functions, did not change significantly. Additionally, this study was limited to results obtained from a single day’s activity over about 2 h, and, thus, can only confirm a short-term effect. The study was also limited by the paucity of cross-sectional research to corroborate its effects. Moving forward, a more detailed examination of the beneficial health effects of forest bathing is required, through the establishment of control groups and the implementation of overnight forest bathing sessions.

## Conclusions

The results of this study suggest that a session of approximately 2 h of forest bathing as part of a 1-day outing in a forest environment can lead to improvements in physiological and psychological health in people of working age, as demonstrated by the decrease in blood pressure and the alleviation of negative psychological parameters after forest bathing.

Moreover, participants with depressive tendencies showed a greater improvement in many of the POMS items after forest bathing compared to those who did not display depressive tendencies. This outcome is evidence that a 1-day forest bathing activity was particularly effective at enhancing the psychological wellbeing of working age people with depressive tendencies.

## Abbreviations

A-H: Anger-Hostility
C: Confusion
DBP: Diastolic blood pressure
D-D: Depression-Dejection
F: Fatigue
HF: High frequency
LF: Low frequency
MCS: Mental component summary
PCS: Physical component summary
POMS: Profile of mood states-brief form
PR: Pulse rate
SBP: Systolic blood pressure
T-A: Tension-Anxiety
TMD: Total mood disturbance
V: Vigor
